# Matching actual treatment with patient administration-route-preference improves analgesic response among acute low back pain patients—a randomized prospective trial

**DOI:** 10.1186/s13018-020-01594-w

**Published:** 2020-02-27

**Authors:** Adi Shani, Michal Granot, Gleb Mochalov, Bennidor Raviv, Nimrod Rahamimov

**Affiliations:** 1 Spine Surgery Unit, Galilee Medical Center, Naharia, Israel; 2 Dept. of Nursing, Faculty of Social Welfare and Health Sciences, University of Haifa, Haifa, Israel; 3 Dept. of Orthopedics B, Galilee Medical Center, Naharia, Israel; 4 Bar-Ilan University Medical School, Safad, Israel; 5 Dept. of Emergency Medicine, Galilee Medical Center, Naharia, Israel; 6 Dept. of Orthopedics B and Spine Surgery, Galilee Medical Center, Naharia, Israel

**Keywords:** Back pain, Placebo, Patient preference, Individualized medicine, Administration route preference

## Abstract

**Background:**

Accommodating a patient’s treatment preference has been reported to promote greater responsiveness and better clinical outcomes. The effect of administration route preference (ARP) on the individual analgesic response has not been extensively examined to date. This study aimed to investigate whether ARP-matched treatment, i.e., individualized intramuscular (IM) or oral (PO) analgesic administration according to patient choice, would increase the analgesic effect.

**Methods:**

In this prospective randomized study, we collected 38 patients with acute low back pain (aLBP) presenting at the emergency room of the Galilee Medical Center (Naharia, Israel) and asked them to report their ARP for analgesics. Regardless of their reported preference, they received either PO or IM diclofenac according to the treating physician’s preference. Pain intensity was self-reported using the numeric pain score (NPS) before and during the first hour after drug administration.

**Results:**

Both groups receiving PO or IM administration reported similar initial pain on admission, (NPS 8.63 ± 1.5 and 8.74 ± 1.6, respectively) and the same magnitude of pain reduction. However, patients who received the drug in their desired route (oral or injection) had a significantly greater reduction in pain levels (4.05 ± 2.8) as compared with patients who received the undesired route (2.08 ± 1.8), *p* < 0.05.

**Conclusions:**

These findings support the hypothesis that individualized ARP-matched treatment in aLBP improves therapeutic outcomes, although further studies with larger cohorts are needed.

## Background

The American Pain Society guideline #11 on management of postoperative pain advocates avoiding painful administration of analgesic therapy [1], thus favoring oral administration over intramuscular injections. As some patients prefer injections and perceive them as being more beneficial than oral therapy [2], adhering to the guideline can come in contrast with the patient-centered trends of recent years and the attempt to individualize treatment [3, 4].

Fosnocht et al. [2] have established that most patients in the emergency department will have an administration route preference (ARP), 66% preferring oral medications (PO), 15% intramuscular (IM), and 19% intravenous (IV). Lindheim et al. [5] in their meta-analysis found a statistically significant effect of shared decision-making and treatment choice on treatment satisfaction, increased completion rates, and clinical outcome.

Greater satisfaction and improvement are assumed to operate through mechanisms attributed also to the placebo response, i.e., positive expectation and conditioning through past experience and beliefs [6]. It is currently accepted that the placebo response refers to the physiological processes experienced following the recognition of being treated, and not as a result of the inert procedure in itself [3]. Therefore, many medical treatments or medical rituals themselves may include a component of placebo response [7].

Prior experience, knowledge, and beliefs that shape positive expectations and conditioning are known to be key components in the placebo response [7] and are also involved in the formation of treatment preference [8], wherein preference can relate to both the type of medication and the mode of its delivery. While medication preference has been studied in this context in the past, the individualized ARP and analgesic ARP have been investigated far less [2, 9, 10].

Our research hypothesis was that the ARP itself is a therapeutic ritual which can encompass key components of the placebo effect (i.e., expectation and conditioning). Thus, administering the same medication in the preferred ARP will increase the analgesic perception, essentially, “if you believe a certain ARP is better, it will be better for you.” The purpose of this study was to investigate our hypothesis that an additive effect will be observed when an analgesic is received in a patient’s ARP, as compared to the same therapeutic agent when received in an undesired method.

To test our hypothesis, patients with acute low back pain (aLBP) were studied, as it is a distinct entity with clear diagnostic criteria and commonly encountered in orthopedic practice. It is generally agreed that there is no known specific etiology and that virtually all cases will resolve with time, regardless of treatment [11, 12]. Current guidelines profess aLBP needs no workup, and treatment is centered on pain relief and patient reassurance [11, 12].

Diclofenac is commonly used for the treatment of aLBP and other types of acute musculoskeletal pain. It is effective and can be administered both orally and intramuscularly to the gluteal muscles [13], making it useful for the purpose of this study. Intra-gluteal injections do have inherent complications such as sciatic nerve or vascular injury, injection site pain, myonecrosis, and infection [14], but they are less common in adults and are a trade-off to complications common to other administration routes [13, 15]. This study did not investigate injections into spinal structures, at times utilized in chronic low back pain (CLBP) or radicular syndromes.

## Methods

This was a randomized prospective case series performed at the emergency room of the Galilee Medical Center (Naharia, Israel), during evening shifts (15:00 to 23:00) on April and May 2019.

*Included* were literate adults,18 to 80 years old, who presented with acute low back pain (aLBP) defined as non-radicular pain, located in the lumbar spine, of a 1-month duration or less, with no so-called “red flags” suggesting severe organic pathology [11].

*Excluded* were patients diagnosed differently or having a known sensitivity to diclofenac.

Patients, referred to our emergency department for acute low back pain, were enrolled by one of the authors (either SA or MG) and treated by the resident orthopedic surgeon. They were asked for their consent to participate in the study and report their ARP (oral administration vs. intramuscular injection) and their initial numeric pain score (NPS, scale of 0–10).

The NPS was chosen as it is a well-established validated measure for self-reported pain in low back pain [16]. The patient is requested to give a numeric value that corresponds to a 0–10 scale where “0” means “no pain at all” and “10” signifies “worst pain imaginable”. A change of over 1.9 is considered to be the minimal clinically important difference [16].

All patients received a single dose of either intra-gluteal diclofenac 75 mg (Abitren® Teva 75 mg/3 ml, Israel) or 100 mg diclofenac, orally (Betaren®100 SR, Dexel, Israel). The dosage differences were in accordance with the recommendations in the literature to adjust for differences in the absorption rate between the digestive system where only 65 to 75% of the active ingredient reaches the bloodstream [17] and the muscular tissue. Diclofenac appears to be completely absorbed when given as a suspension, capsule, or tablet. The administration of a diclofenac solution shows rapid absorption with Cmax being attained within 10 to 40 min [18].

As either IM or PO diclofenac are routinely used at our emergency department in the treatment of aLBP, allocation to receive one or the other (regardless of the initial analgesic ARP) was done according to the usual choice of the treating on-call resident physician, blinded to the ARP noted by the patient. Demographic and medical information was collected using a questionnaire, and NPS (0–10) were collected every 10 min during the first hour following the analgesic administration.

### Statistical analyses

The statistical analysis was performed using IBM SPSS (SPSS Inc., Chicago, IL, USA, version 23).

The patients were divided according to their preference (i.e., IM or PO) and also according to the actual administration route. The term “matched” being patients where the actual administration route matched their preference whereas “non-matched” being patients in which the actual administration route did not match their preference.

Dichotomic or discrete data were described by frequencies and percentages. Continuous variables were described by mean, standard deviation, and range. We compared the groups with parametric tests (chi-square test for the qualitative data and independent-sample *t* test for the quantitative data) and also utilized nonparametric tests (Fisher’s exact for the qualitative data if expectancy < 5 and Wilcoxon rank-sum test if the sample size was small and the variable distribution violated significantly the normal distribution). Both tests, parametric and nonparametric, achieved similar results.

*P* value less than 0.5 was considered a significant result. Two-tailed P values were noted.

## Results

Included were 38 patients who met our inclusion criteria. Twenty were female and 18 male. The mean age was 43.45 years (median 41.5, std 14.32). Twenty-five patients received IM diclofenac and 13 PO. Both groups were identical in their demographic characteristics (Table 1).

**Table 1:** Demographic data according to administration route

	PO		% of total sample	IM		% of total sample	*P* value
	*N* = 13	% of group	34.2	*N* = 25	% of group	65.8	
Age (mean ± sd)	42.8 (± 15.7)			43.48 (± 15.25)			**0.288**
Gender							**0.743**
Female	5	38.5%	13.1	12	48%	31.6	
Male	8	61.5%	21	13	52%	34.2	
Place of birth							**0.108**
Israel	8	61.5%	21.1	23	92%	60.5	
Russia	3	23.1%	7.9	1	4%	2.6	
North America	1	7.7%	2.6	1	4%	2.6	
Africa	1	7.7%	2.6				
Marital status							**0.571**
Single	2	15.4%	5.3	6	24%	15.8	
Married	10	76.9%	26.3	15	60%	39.4	
Divorced	1	7.7%	2.6	4	16%	10.5	
Widowed	0			0			
Formal education							**0.176**
None	1	7.7%	2.6	2	8%	5.3	
Grade school	1	7.7%	2.6	3	12%	7.9	
High school	6	46.2%	15.8	10	40%	26.3	
Academic	4	30.8%	10.5	2	8%	5.3	
Vocational	1	7.7%	2.6	8	32%	21	

The original intention was to collect 30 patients in each group to achieve a power of 81% (based on a 2-tailed independent-sample *t* test, alpha = 5%). Due of the difficulties in recruitment of patients, we had 16 patients in the non-matched group and 22 patients in the matched group and therefore achieved the power of 72% for our 1-tailed hypothesis (based on the required difference as mentioned above and also the final results of our research) (Table 2).

**Table 2:** Patient grouping according to ARP vs. actual administration method

		Administration route preference	
		PO (*n* = 21)	IM (*n* = 17)
Actual administration method	PO (*n* = 13)	A (prefer PO, received PO, *n* = 9)	B (prefer IM, received PO, *n* = 4)
	IM (*n* = 25)	C (prefer PO, received IM, *n* = 12)	D (prefer IM, received IM, *n* = 13)

**Fig. 1:**
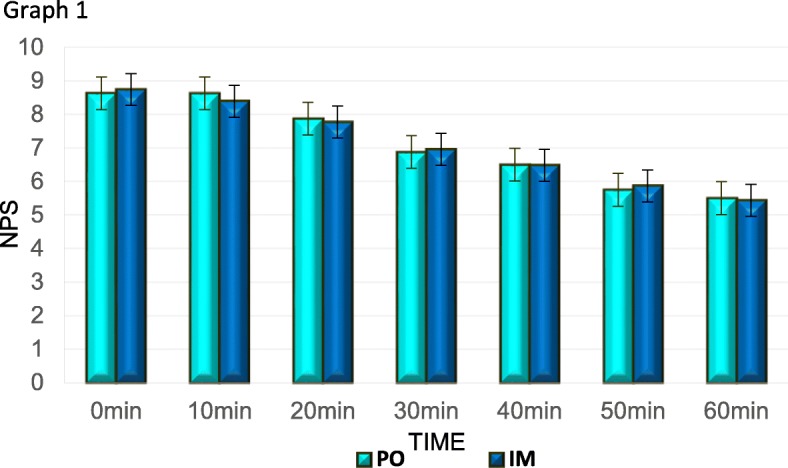
Comparison between pain reduction in patients receiving PO and IM administration. Pain reduction did not show a significant difference between the two groups

No differences were found in mean pain levels between the IM and PO groups, as both groups reported similar severe initial pain, (NPS 8.63 ± 1.5 and 8.74 ± 1.6, respectively) as well as a similar magnitude of pain reduction (Fig. 1, Table 3).

**Table 3:** NPS reduction by administration route

Time from administration (min)	Administration route	*N*	Mean	std deviation	*P*	95% confidence interval	
						Lower	Upper
10	PO	13	8.08	1.706	0.755	− 1.511	1.104
	IM	25	8.28	1.969			
20	PO	13	7.38	1.805	0.777	− 1.611	1.214
	IM	24	7.58	2.125			
30	PO	13	6.62	2.399	0.826	− 1.872	1.503
	IM	25	6.80	2.449			
40	PO	13	6.31	2.496	0.974	− 1.694	1.749
	IM	25	6.28	2.475			
50	PO	13	5.62	2.567	0.908	− 1.918	1.709
	IM	25	5.72	2.638			
60	PO	13	5.31	2.626	0.958	− 2.049	1.945
	IM	25	5.36	2.998			

To further stratify the results, 4 groups of preference were created (Table 2):

A. Patients who preferred and received oral treatment (*n* = 9)
B. Patients who preferred intramuscular injections and received oral treatment (*n* = 4)
C. Patients who preferred oral treatment and received intramuscular injection (*n* = 12)
D. Patients who preferred and received intramuscular injection (*n* = 13)

Since group B was too small to perform statistical analysis, the four groups were re-divided into two groups:

1. Patients who were treated according to their ARP’s—whether oral treatment or intramuscular injection (groups A and D in Table 2, *n* = 22)
2. Patients who received treatment that did not match their ARP’s (groups B and C in Table 2, *n* = 16)

**Fig. 2:**
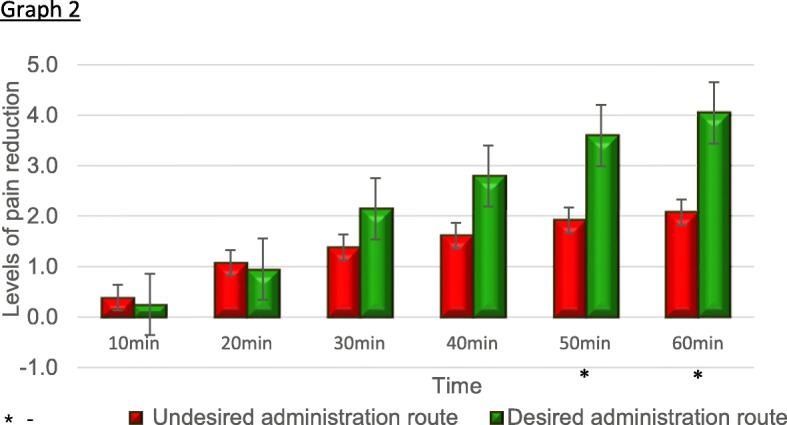
Pain reduction in patients receiving the medication in the desired ARP versus the undesired ARP. The group receiving the medication in their desired ARP showed a significantly better reduction in self-reported pain levels, reaching statistical significance at 50 min post administration

When the NPS reduction was examined according to this grouping, a consistent trend of a greater reduction in pain levels was observed in the group of patients treated according to their preference compared with those who received treatment that did not match their preference at 20 min post-administration. Statistical significance was reached at 50 min (*P* = 0.019) and remained significant at 60 min (*P* = 0.032) (Fig. 2, Table 4).

**Table 4:** NPS reduction by ARP matching

Time from administration (min)	Matched ARP	*N*	Mean	std deviation	*P*	95% confidence interval	
						Lower	Upper
10	No	16	.3125	.79320	0.710	− 0.37672	0.54718
	Yes	22	.2273	.61193			
20	No	15	.9333	1.09978	0.953	− 0.81030	0.85878
	Yes	22	.9091	1.30600			
30	No	16	1.3125	1.25000	0.182	− 1.82649	0.36058
	Yes	22	2.0455	2.05814			
40	No	16	1.5000	1.41421	0.061	− 2.42238	0.05874
	Yes	22	2.6818	2.12438			
50	No	16	1.8750	1.40831	0.019	− 2.88515	− 0.27395
	Yes	22	3.4545	2.52091			
60	No	16	2.1250	1.66833	0.032	− 3.31843	− 0.15885
	Yes	22	3.8636	2.76535			

**Fig. 3:**
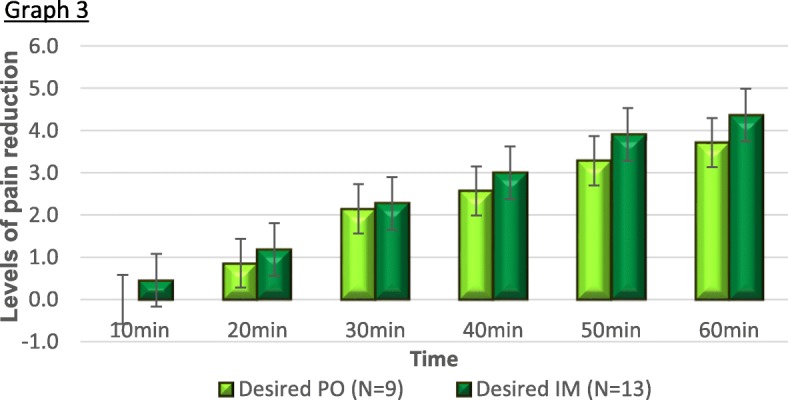
Pain reduction in patients receiving the medication in their desired ARP. Both groups showed a similar reduction of self-reported pain levels regardless of administration route

When divided into groups according to the administration method, the intramuscular group was larger than the oral group. To rule out the possibility that the effect was due to the injection itself, the group of patients who received IM medication according to their preference (*n* = 13) was compared to the group of patients who received oral medication according to their preference (*n* = 9) (Fig. 3, Table 5) with no difference found between these two groups, suggesting the injection itself was not the cause of this finding.

**Table 5:** NPS reduction by administration route in patients that received their ARP

Time from administration (min)	Desired and received (matched) ARP	*N*	Mean	std deviation	*P*	95% confidence interval	
						Lower	Upper
10	PO	10	.0000	.00000	.114	− 0.94191	0.10858
	IM	12	.4167	.79296			
20	PO	10	1.0000	1.15470	.774	− 1.02607	1.35940
	IM	12	.8333	1.46680			
30	PO	10	2.1000	1.85293	.913	− 1.78306	1.98306
	IM	12	2.0000	2.29624			
40	PO	10	2.6000	2.11870	.874	− 2.09300	1.79300
	IM	12	2.7500	2.22077			
50	PO	10	3.2000	2.39444	.676	− 2.76354	1.83021
	IM	12	3.6667	2.70801			
60	PO	10	3.3000	2.40601	.396	− 3.51790	1.45124
	IM	12	4.3333	3.05505			

In addition to the observed statistical significance, it is also important to note that the difference between ARP-matched and non-matched groups closely matches or exceeds the 2/10 minimal clinically important difference (MCID) in NPS [16]. The mean reduction in pain levels in the non-matched ARP group was 1.92 after 50 min and 2.08 after 60 min. In the matched ARP group, the pain reduction was more pronounced, with a reduction of 3.6 and 4.05 points, respectively (at 50 min (*p* = 0.019)and at 60 min (*p* = 0.032)).

## Discussion

It has long been established that patients have distinct preferences, including administration route preferences, and complying with individual preference can lead to greater patient satisfaction and treatment outcomes [2, 5, 8–10, 13, 19, 20]. Our study aimed to find whether matching ARP for the same analgesic medication can result in better outcomes. We have found that ARP matching, 1 h post treatment, leads to an NPS reduction difference of more than two points (or twofold) better reduction when compared to non-ARP-matched treatment, although initial pain levels were similar.

Intramuscular administration may augment pain reduction due to activation of descending pain inhibition, as by the pain-inhibits-pain phenomena, in which the pain evoked by the needle activates descending pain pathways [21–23] suppressing pain. In addition, the “needle effect” [24, 25], in which the injection trauma itself is thought to instigate an analgesic response, may be part of the results found. To rule this out, patients who received their desired ARP (whether it be PO or IM) were compared, showing no difference.

As there was no difference in the analgesic effect between the administration method groups when given according to the ARP’s, it can be assumed that it is not the administration method per se which led to the significance of the findings, but rather the ARP matching.

In addition to the observed statistical significance, it is also important to note that the difference between ARP-matched and non-matched groups exceeded the 2/10 accepted MCID in NPS for back pain [16] or the 15/100 for mixed acute pain in the emergency department [19].

Although shared decision-making has been well established as beneficial in patient compliance, satisfaction, and clinical outcomes [3, 6, 8, 9, 26, 27], we have not found in the literature previous studies focusing on the specific effect of ARP matching on pain reduction. Preference in general is associated with prior knowledge, attitudes, belief, learning, and conditioning and therefore expresses an expectation of a positive effect of the preferred treatment. All of these have been associated with endogenous processes that promote recovery [28]. Furthermore, expectation learning and conditioning are known to be key components of the placebo phenomenon [29–31]. It is therefore possible that the analgesic improvement demonstrated in this study also involves a placebo response.

Our findings also support shared decision-making regarding treatment and emphasize the importance of considering other factors that shape the experience of pain other than the physiological aspect [32, 33].

Our study has several limitations; the most notable is the small sample size that limited the statistical comparisons which could be performed.

Since the treatment given in this study was mostly by orthopedics residents on call, another limitation is the variability of caregivers, the amount of time they had to interact with the patients, and their personal ability to convey compassion and trust—key elements in clinical practice success.

Due to these limitations, we cannot argue that the adjustment of the ARP to the treatment given, regardless of the type of treatment or method of administration, is solely responsible for the outcome. To establish this, further studies with larger samples are needed.

## Conclusions

The findings of our current study support previously published data that treatment based on patient preference improves therapeutic and analgesic outcomes [20, 26, 27] and adds the administration route preference as an additional consideration for the practitioner when prescribing analgesic treatment.

## Data Availability

All data is provided in the manuscript and tables. There are no supplementary data or material.

## Abbreviations

aLBP: Acute low back pain
ARP: Administration route preference
IM: Intramuscular
NPS: Numeric pain score
PO: Per os
